# A Workability Characterization of Innovative Rubber Concrete as a Grouting Material

**DOI:** 10.3390/ma15155319

**Published:** 2022-08-02

**Authors:** Yi Lu, Cong Li, Xiaoyu Zhang, Xiangyun Huang, Zhongyin Zhao

**Affiliations:** 1 School of Civil Engineering, Guangzhou University, Guangzhou 510006, China; luyi@gzhu.edu.cn; 2 Earthquake Engineering Research and Test Center, Guangzhou 510006, China; 2112016162@e.gzhu.edu.cn (C.L.); eertchxy@gzhu.edu.cn (X.H.); 3 Liaoning VVSUN Waterproof Insulation Engineering Co., Ltd., Guangzhou 510006, China; shuimojing2022@163.com

**Keywords:** grouting material, shield tunnel, rubber, PVA fiber, UCS

## Abstract

The aim of this study is to assess the workability of an innovative rubber concrete to fill in the gaps in a shield tunnel construction. This grouting material includes porous sand, PVA (polyvinyl alcohol) fiber, cement, and rubber. One advantage of the material is its high toughness, which is good for the postfailure stage of an earthquake event. Evaluations were carried out on the physical properties in terms of the slurry density, consistency, fluidity, bleeding rate, consolidation shrinkage rate, setting time, and unconfined compressive strength (UCS) (i.e., 3 and 28 days). The toughness index was also introduced to evaluate the performance at the postfailure stage. The results demonstrate that the toughness index will increase as the rubber content increases. It increases from 1.0 at 0% to 1.7 at 80% rubber content (28 days’ curing) and from 1.2 at 0% to 2.2 at 80% rubber content (3 days’ curing). The increase in fiber content and fiber length will also increase the toughness index as the fiber will enhance the tensile strength of the matrix. The results show that when the fiber content increases from 0% to 1%, the toughness index increases from 1 to 7 (28 days’ curing) and from 1.1 to 10 (3 days’ curing). Similarly, if the fiber content or fiber length is above the optimum level, the UCS of the material will be compromised. The optimum fiber content is 0.8%, and the optimum fiber length is 6 mm to 9 mm. This study suggests that the balance of physical properties should be considered in designing a satisfactory grouting material based on the specific purpose of the engineering practice.

## 1. Introduction

Due to automation, the possibility of rapid construction, and a lower impact on the ground, shielding is a widely used method in tunnel construction [1]. During the excavation process, a gap will be induced between the tunnel segment and surrounding soil [2]. This gap should be filled in to avoid geotechnical hazards (e.g., deformation, collapse) from the surrounding soils [3]. Usually, the shield machine pumps the grouting material to fill the gap during the excavation process. After some time, the grouting material will form a solid layer between the tunnel segment and soils [4]. Many studies have been conducted to find eco-friendly materials for both structural and thermal purposes [5,6].

The commonly used grouting materials can be divided into three categories: lime-based grouting materials, cement-based grouting materials, and double-liquid grouting materials [7,8,9,10]. Lime-based grouting materials have a long setting time, low strength, low price, and good fluidity. Cement-based grouting materials have medium-level early strength and high-level late strength, but the stability is usually poor. The double-liquid grouting materials have high strength, high cost, and a complex construction process. The setting time of the double-liquid grouting materials can be controlled and ranges from a few seconds to several hours. Extensive studies have been conducted on grouting materials to enhance the tunnel construction, mechanical properties, durability, economic efficiency, etc. [1,7,8,11,12,13,14,15,16,17]. There is no doubt that these studies have made a significant contribution to knowledge, but practical problems such as tunnel segments floating in the grouting materials, low resilience to earthquakes, and metro lines causing vibration of the structure have not been solved [18,19,20,21,22].

The partial replacement of aggregate with rubber in conventional concrete to form a rubber concrete has also been studied extensively [23]. Both the physical and chemical properties of rubber concrete have been addressed in terms of workability, static and dynamic properties, durability, thermal dynamics, sound insulation, etc. [24,25,26]. The conclusions of these studies have shown that adding rubber to the concrete can significantly improve the vibration transmission, toughness, impact resistance, damping ratio, etc. [27,28,29,30]. However, the addition of rubber will also compromise the compressive strength, tensile strength, and elastic modulus compared with the conventional concrete [31,32,33].

Tunnel construction will be subject to seismic or impact load and may suffer damage. From the aforementioned studies, we know that conventional rubber concrete provides a possible solution for minimizing the damage. Additionally, for tunnel construction, grouting the rubber concrete in the liquid form is an efficient method to mitigate damage. However, to the best of the authors’ knowledge, the workability for grouting material assessment is insufficient. Based on the above studies and inspired by porous materials (e.g., loosely deposited sand, foam concrete) [34,35,36,37,38], this study aims to assess grouting material by introducing porous sand and fiber to rubber concrete to fill in the gap between the tunnel segment and the surrounding soils. The purpose of adding rubber to concrete is to minimize the seismic or impact load on the filling material. The purpose of adding fiber is to enhance the tensile strength of the rubber concrete [30,39,40,41].

## 2. Materials

**Figure 1:**
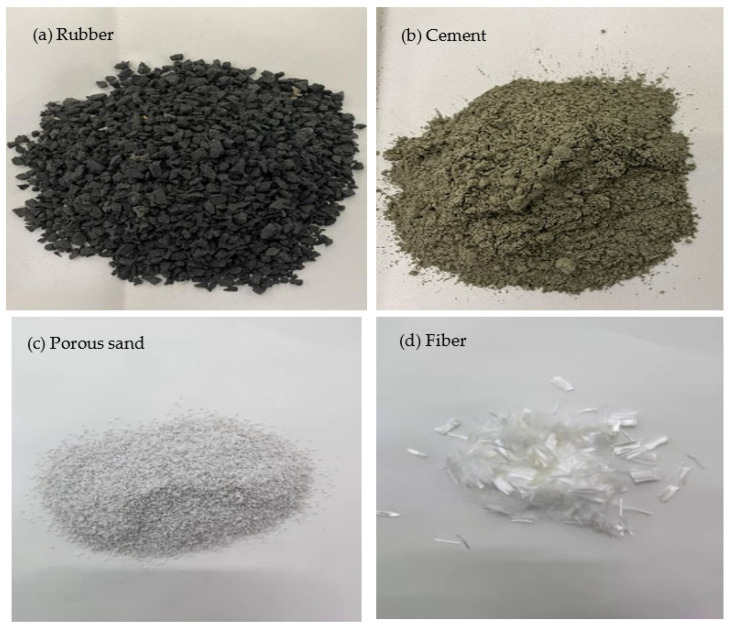
Profiles of rubber, cement, porous sand and fiber.

The raw materials used in this study include rubber, cement, porous sand, and fiber (Figure 1). The basic properties of these materials are shown in Table 1, Table 2 and Table 3. The particle size of the rubber is between 2 and 4 mm, and the bulk density is 0.716 g/cm^3^.

Table 4 lists the test conditions in this study; each test included two identical specimens for repeatability. The average was taken in the following analysis. Group A was used to evaluate the different rubber content influences. Based on A4, Group B was used to assess the effects of the fiber content on the mechanical behavior of the grouting material. Based on B1, Group C was used to investigate the effects of fiber length on the solidified mechanical behavior [42,43]. Similar tests and methods can be found in the literature [42,43]. Each test No. has two identical specimens, and the average was taken for analysis. Group B and C are based on A4.

## 3. Specimen Preparation and Experimental Program

**Figure 2:**
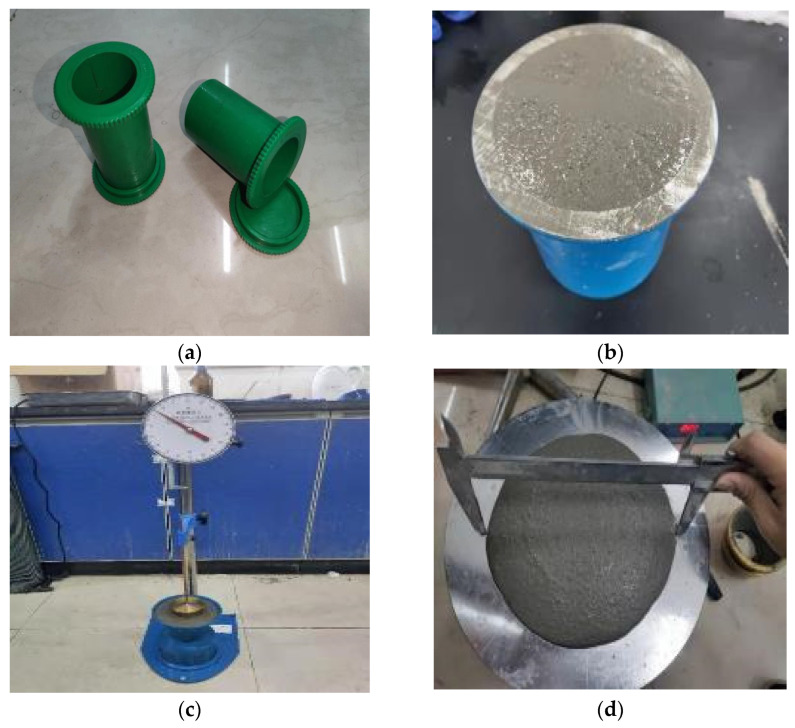

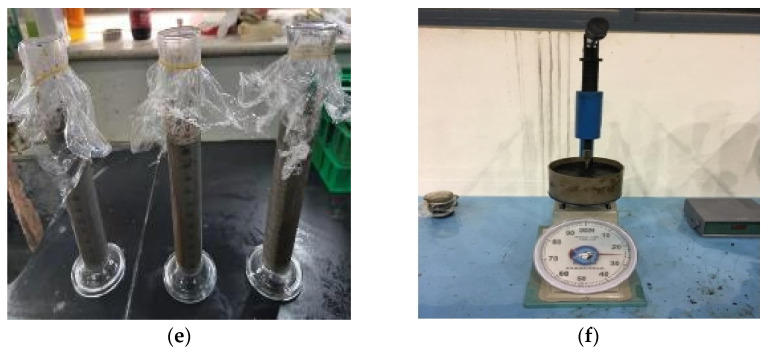
Profiles of the different physical characterizations. (**a**) Mold for specimen; (**b**) slurry density test; (**c**) consistency test; (**d**) fluidity test; (**e**) bleeding rate and consolidation shrinkage rate test; (**f**) setting time test.

The target dry masses of cement, porous sand, rubber, and fiber were mixed manually in a conventional mixer to ensure a relatively good distribution of these materials with the cement (Table 4). Subsequently, the target mass of water was added to the mixture in the mixer by the spraying technique at 120 RPM rate. The mixing process was conducted after 15 min to ensure a homogeneous state. The room conditions were controlled at 20 ± 1 °C. A similar mixing approach can be found in other studies [44,45]. After proper mixing, part of the slurry in the mixer was poured into the mold (i.e., 50 mm in diameter and 100 mm in height) on a vibration table. The mold was smeared with silicone oil to minimize the friction for demolding (Figure 2a). The vibration process at 1 Hz was expected to remove the air trapped in the specimen on the vibration table [42,43,46,47]. Finally, both ends of the specimen in the mold were leveled and carefully wrapped with vinyl foam. The sealed specimens in the mold were then transferred and stored in a controlled environment (i.e., 95% humidity and 20 ± 1 °C) after 3 and 28 days for the UCS test. The rest of the slurry in the mixer was evaluated for other physical properties as shown in the following section. At the demolding stage, the mold was carefully dissembled so the specimen could be taken out with sufficient stiffness.

It needs to be highlighted that the total volume of aggregate (the total volume of rubber and porous sand) was constant in Group A (Equation (1)), although the rubber content and porous sand content in Group A increased and decreased (Table 4), respectively. The mixing ratios in Groups B and C to investigate the fiber content and fiber length were based on A4.

(1) $$\frac{m_{R}}{\rho_{R}}+\frac{m_{PS}}{\rho_{PS}}=\;V_{T},$$

where $m_{R}$ is the mass of rubber; $\rho_{R}\;$ is the bulk density of rubber; $m_{PS}$ is the mass of porous sand; $\rho_{PS}$ is the bulk density of porous sand; and $V_{T}$ is the total volume of aggregate.

## 4. Workability Evaluation

### 4.1. Slurry Density Test

The slurry density test was conducted based on JGJ/T70-2009 [46]. The key steps of the test were to fill the container with slurry and then remove the air from the slurry by tapping the container wall with a hammer (Figure 2b). Then, the slurry density was the ratio of the mass of slurry to the volume of the container.

### 4.2. Consistency Test

The consistency test was conducted based on JGJ/T70-2009 [38]. A cone-shaped cup was first filled with slurry, and then we adjusted the drop level to make contact with the slurry surface (Figure 2c). An initial dial reading was taken. Subsequently, the drop was released to freely fall into the slurry. The final dial reading was taken after 10 s. The consistency of the slurry was defined as the difference between these two readings.

### 4.3. Fluidity Test

The fluidity test was carried out in accordance with GB/T 2419-2005 [48]. The slurry was first poured into the mold, and then we removed any air trapped in the slurry by tapping the mold. Then, the slurry was poured onto a fluidity vibration table to complete 25 vibrations within 25 s. The two diameters of the slurry in the perpendicular direction were measured by vernier calipers (Figure 2d). Finally, the average of the two diameters was taken as the fluidity.

### 4.4. Bleeding Rate Test

The bleeding rate test was carried out in accordance with T/CECS 563-2018 [49]. A 250-mL cylinder was used for this test (Figure 2e). The slurry was poured into the cylinder to the 245 ± 5 mL level. The initial slurry surface level, ***a*_0_**, was recorded after 1 min. After three hours’ solidification in sealed conditions, ***a*_1_** (i.e., the water level of the slurry) and ***a*_2_** (i.e., the slurry level after solidification) were recorded. Consequently, the bleeding rate in 3 h can be calculated as in Equation (2) [49]:

(2) $$BR_{3h}=\frac{a_{1}-a_{2}}{a_{0}}\times 100\%,$$

where $BR_{3h}$ is the 3 h bleeding rate; ***a*_0_** is the initial slurry surface level; ***a*_1_** is the water level of the slurry after a 3 h solidification; and ***a*_2_** is the slurry level after a 3 h solidification.

### 4.5. Consolidation Shrinkage Rate Test

Similar to the bleeding rate test, a consolidation shrinkage rate test was carried out in accordance with T/CECS 563-2018 and using the same 250-mL cylinder (Figure 2e) [49]. The slurry was poured into the cylinder in sealed conditions. The consolidation shrinkage rate was calculated as shown in Equation (3) [49]:

(3) $$S=\frac{h_{1}-h_{2}}{h_{1}}\times 100\%,$$

where ***S*** is the consolidation shrinkage rate; $h_{1}$ is the initial slurry surface level; and $h_{2}$ is the final slurry level after 3 h.

### 4.6. Setting Time Test

The slurry setting time test (Figure 2f) was carried out according to JGJ/T70-2009 at a room temperature of 20 ± 2 °C [46]. The slurry was first poured into a cone-shaped cup, and then a 30-mm^2^ needle was dipped 25 mm into the slurry to measure the penetration resistance (***fp***). As determined from Equation (4), ***f_p_*** is the key parameter used to obtain the setting time test [46]. Once ***f_p_*** is 0.7 MPa, the time spent is defined as the setting time:

(4) $$f_{p}=N_{p}/A_{p},$$

where $f_{p}$ is the penetration resistance; $N_{p}$ is the friction against penetration 25 mm into the slurry; and $A_{p}$ is the cross-sectional area of the needle (30 mm^2^ in this case).

### 4.7. Unconfined Compressive Strength (UCS) Test

**Figure 3:**
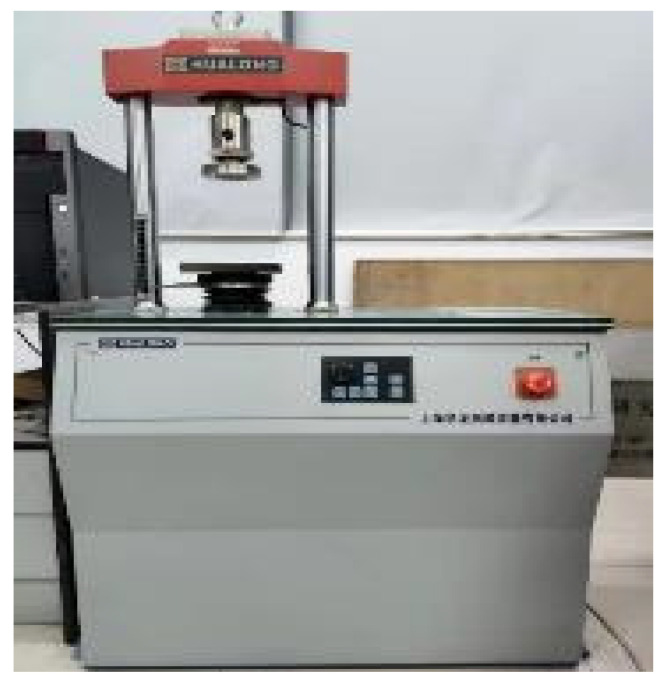
Profile of UCS test.

UCS tests (Figure 3) were carried out under a loading rate of 1 mm/min according to GB/T 50266-2013 [50]. The displacement and stress were both recorded with a computer. Curing times of 3 and 28 days were used for the UCS test.

## 5. Results and Discussions

### 5.1. Verification of Repeatability

**Figure 4:**
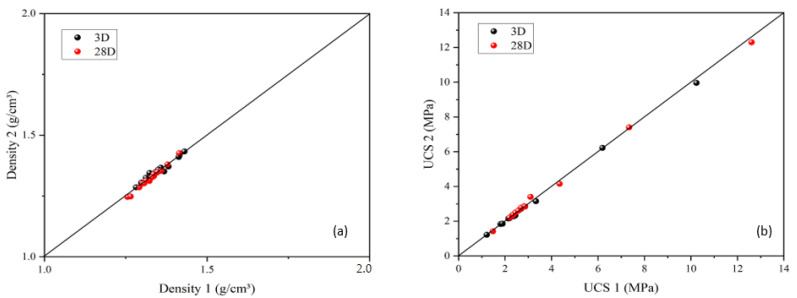
Verification of each specimen in terms of slurry density (**a**) and UCS (**b**).

When the curing process was finished, each specimen was prepared for a UCS test by demolding. As mentioned in the previous section, each test condition included two identical specimens to reduce the experimental error. Therefore, the repeatability was verified in terms of specimen density and UCS. As can be seen from Figure 4, the two measured densities and UCS were almost the same, as these points are on the 1:1 gradient. Based on Figure 4, it can be concluded that the results obtained in this study are acceptable.

### 5.2. Effect of Rubber and Porous Sand on Aggregate

**Figure 5:**
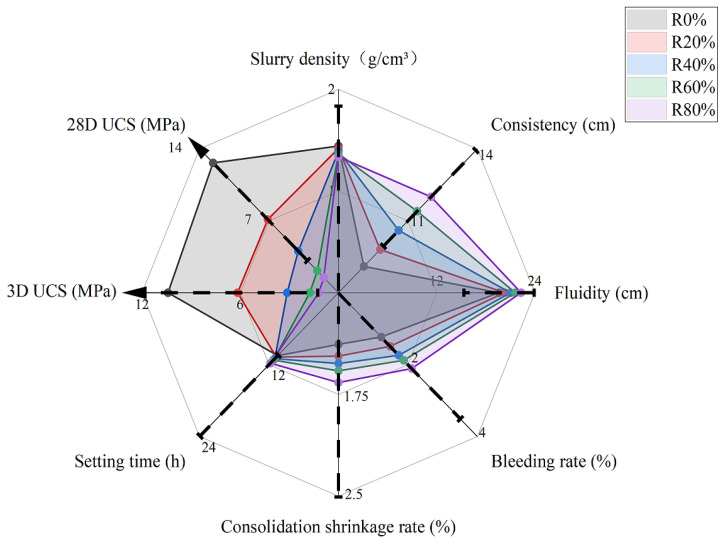
Performance with different rubber and porous sand content without fiber.

The general behavior of the physical indices for the workability is shown in Figure 5. When the rubber content increased in the UCS for 3 and 28 days, the slurry density reduced, but the consistency, fluidity, bleeding rate, setting time, and consolidation shrinkage rate increased. It was also observed that some of the test conditions were outside of the recommended requirements, e.g., R60% and R80% were outside of the lower limit of 28 UCS, while R0% and R20% were outside of the lower limit of consistency. However, most testing conditions fell within the recommended requirements. For this study, R40% was the best mixing ratio for Group A, R0% and R20% were outside of range in terms of consistency, and R60% and R80% were outside of range in terms of the 28D UCS.

**Figure 6:**
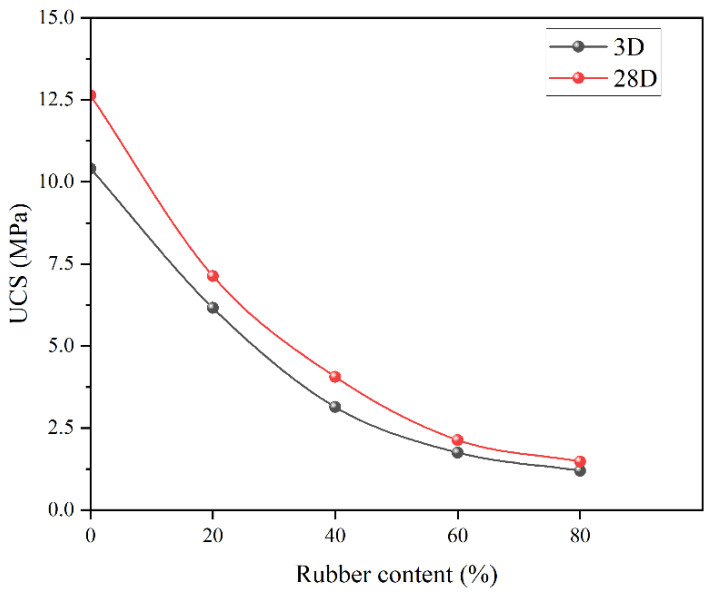
UCS for different rubber content without fiber.

Figure 6 shows the UCS change against rubber content. As can be seen, there was a significant drop from 0% to 60% since UCS drops from 12.5 MPa to 2.5 MPa at 28 days and from 10.5 MPa to 2.3 MPa at 3 days. The tendency became insignificant for the rubber content between 60% to 80% as UCS dropped from 2.5 MPa to ~2.1 MPa at 28 days and from 2.3 MPa to ~1.7 MPa at 3 days. This is consistent with other studies [51,52,53]. When there was an external load acting on the rubber concrete, the rubber deformed and the interface shear resistance were lower with concrete, leading to the mixture failing. It was observed that UCS for 28 days at 60% was just below the recommended requirement, which is 2.5 MPa (Table 5).

**Figure 7:**
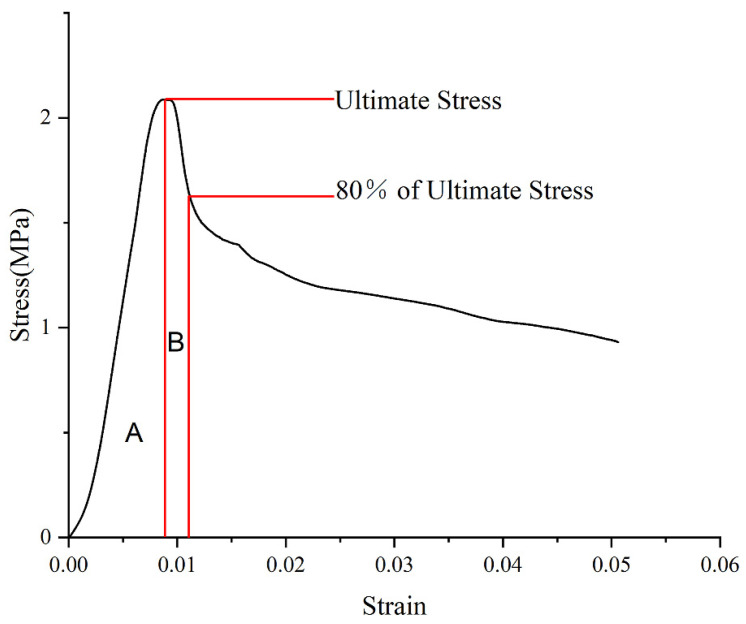
Calculating the toughness index.

**Figure 8:**
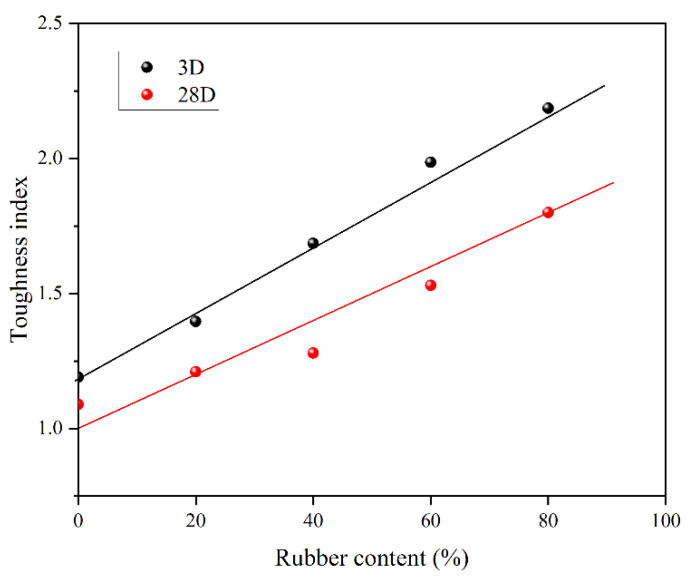
Toughness indices for different rubber contents without fiber.

However, this increase in the rubber content will contribute to a significant increase in the toughness index Equation (5) at the postfailure stage (Figure 7), as the toughness index rises for both 3 and 28 days (Figure 8). This will delay the collapse of the material after the failure. Additionally, the toughness index for 3 days is higher than that for 28 days, due to the low brittleness of the shorter curing time. So, we should have more concerns about the material with higher brittleness.

(5) $$T_{i}=\frac{T_{80\%}}{T_{100\%}},$$

where $T_{i}$ is the toughness index; $T_{100\%}$ is the area integral to the ultimate stress; and $T_{80\%}$ is the integral area from ultimate stress to 80% of ultimate stress at the postfailure stage (Figure 7).

It also needs to be emphasized that the advantage of adding porous sand instead of solid sand is to introduce a porous property to the matrix, so the grouting material after solidification is able to be compressed to a relatively large deformation. This mechanism and concept are consistent with other porous materials such as loose sand deposits or foam concrete [34,35]. However, more experiments are necessary to confirm this point.

### 5.3. Effect of Fiber Content

**Figure 9:**
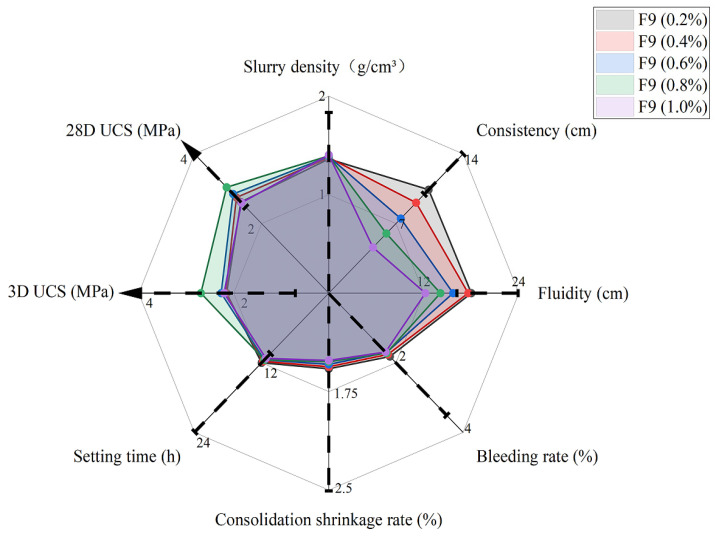
Performance with different fiber contents at rubber content of 60% and 9 mm in fiber length.

Based on Group A4, the effect of fiber content (with a length of fiber of 9 mm) on the workability was assessed. It can be observed from Figure 9 that most data were within the required range, except for 0.4–1.0% of consistency and 0.6–1.0% of fluidity. This indicates that slurry is more difficult to pump to fill the gap in engineering practice when it has a high fiber content. The boundary fiber content should be determined according to a specific mixing ratio.

**Figure 10:**
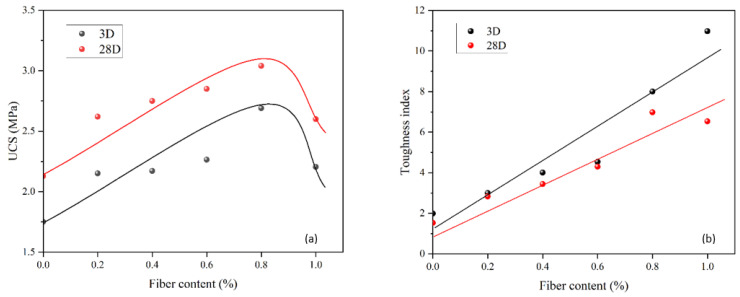
UCS for different fiber contents (**a**) and toughness indices for different fiber contents (**b**) at rubber content of 60% and 9 mm in fiber length.

Figure 10a shows the UCS against different fiber contents for the 3- and 28-day specimens. The UCS tendency first increases to the optimum level and then starts to drop. In this study, the optimum fiber content was about 0.8% for the UCS of 3 MPa at 28 days and 2.7 MPa at 3 days. Concrete is usually vulnerable to resistant tensile stress, so adding fiber overcomes this limitation. When there is stress acting on the concrete, it will be transferred to the fiber and resisted by the tensile strength. This additional resistance will delay the development of microvoids in the concrete, increasing the UCS of the concrete matrix. However, if the fiber content is above the optimum level, UCS starts to drop as more fiber is weak against the compressive stress. Therefore, the optimum fiber content should be determined to avoid a compromise in UCS. Similarly, the toughness index in Figure 10b increases as the fiber content increases, and specimens cured for 3 days always have a higher value than those cured for 28 days due to the lower brittleness. At the postfailure stage, fiber will play a significant role in filling cracks and eventually delays the collapse of the material. This will allow the material to have a high residual strength despite large deformations.

Figure 10 shows that adding fiber to the optimum value of 0.8% will increase UCS due to the strong interface shearing resistance of fiber to the concrete, but a larger fiber content is not always preferred for the grouting material as the fluidity (i.e., 0.2% and 0.4% are good) and consistency (i.e., 0.2% is good) will be compromised (Figure 9), and the mixing becomes difficult. This means that a balance of properties should be considered to determine the contents before application; the best fiber content is about 0.2% in this study.

### 5.4. Effect of Fiber Length

**Figure 11:**
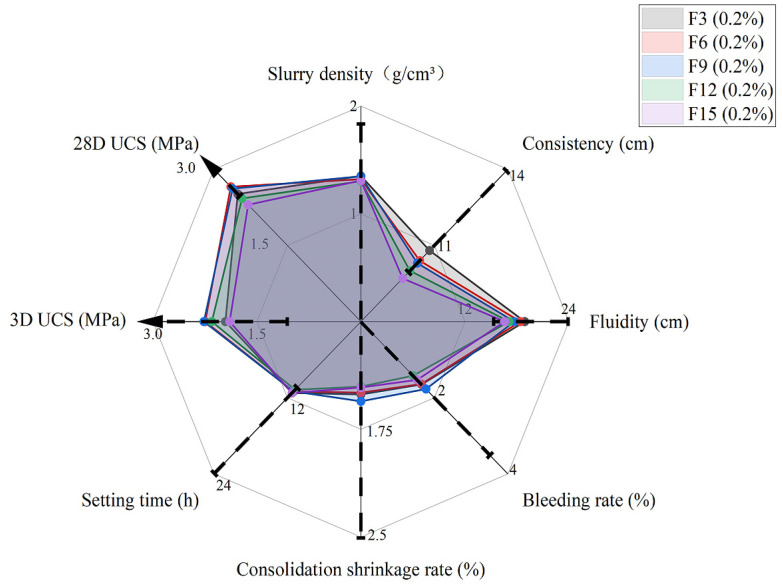
Performance with different fiber lengths at rubber content of 60% and fiber content 0.2%.

**Figure 12:**
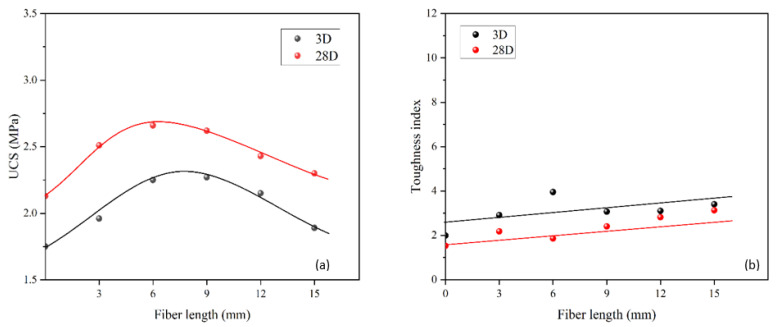
UCS for different fiber lengths (**a**) and toughness indices for different fiber lengths (**b**) at rubber content of 60% and fiber content 0.2%.

The same tests were conducted for fiber length to evaluate the workability. Comparisons were also made with the recommended requirements (Figure 11). As can be seen from Figure 11, F15 (0.2%) was outside of the lower range for consistency, and the fluidity decreased when the fiber length increased. Moreover, as the fiber length increased, UCS for 3 and 28 days first increased to the optimum level and then started to drop (Figure 12a), which was the same pattern as for the fiber content behavior (Figure 10a). Usually, a longer fiber will increase the contact resistance between the fiber surface and the concrete matrix, so the connection in terms of tensile strength will be stronger, which is beneficial for concrete under an external load. However, if the fiber length is above the optimum level, it will make the mixing process more difficult and cause the fiber distribution in the concrete matrix to be uneven. This will eventually lower the UCS due to the poor mixing. In this study, the optimum fiber length is between 6 mm and 9 mm for 3 days and about 6 mm for 28 days. Similarly, the toughness index increased slightly as the fiber length increased due to the good tensile strength of the fiber at the postfailure stage. However, the improvement of the toughness of the fiber length was insignificant with the same fiber content (i.e., Figure 10b compared with Figure 12b). Therefore, we should be careful to choose a longer fiber as the UCS will drop significantly compared with a small increase in the toughness index. Thus, only F15 (0.2%) was outside of range in terms of consistency, and 6–9 mm was the optimum level: Based on Group C, we found that F6 (0.2%) and F9 (0.2%) were the best lengths for workability.

## 6. Conclusions

A series of experimental tests were conducted to evaluate the physical properties of an innovative grouting material by introducing porous sand and fiber to rubber concrete. The toughness index of the material was improved, which was beneficial for the postfailure stage. It was found that increasing the fiber content enhanced the mechanical properties (i.e., UCS and toughness index). However, the increasing fiber content compromised the workability of the grouting material (e.g., fluidity). Thus, the best value for fiber content had to be determined experimentally and was found to be 0.2% in this study. Similarly, the fiber length also had an optimum value (about 6 to 9 mm in this study). Above the optimum, the grouting material is less useful in terms of fluidity and makes mixing impossible. Additionally, for fiber lengths up to 15 mm, the toughness index only increased to about 3, while for fiber content up to 1%, the same toughness index increased to about 8. However, the grouting material in this study was based on the particular conditions of load type, rubber, cement, and fiber, so the results obtained are only relevant to this particular engineering application. Studies in terms of sand properties, loading conditions, grouting method, fiber type, etc. are recommended for finding the best general grouting material.

## Figures and Tables

**Table 1:** Composition of the cement given by the supplier.

Composition	Content (%)
CaO	61.11
SiO_2_	24.3
Al_2_O_3_	5.33
Fe_2_O_3_	4.38
SO_3_	2.24
MgO	1.36
Loss on ignition	1.28

**Table 2:** Properties of fiber given by the supplier.

Diameter (mm)	Modulus of Elasticity (MPa)	Tensile Strength (MPa)	Elongation at Break (%)	Density (g/cm^3^)
0.2	15,000	950	27	0.91

**Table 3:** Properties of porous sand given by the supplier.

Size (mm)	Bulk Density (g/cm^3^)	Maximum Absorbed Water (%)
1–1.5	117	43

**Table 4:** Mixing ratios in terms of mass of each test condition.

Test No.	Abbreviation	Water (g)	Cement (g)	Porous Sand (g)	Rubber (g)	Rubber Content (%)	Fiber (g)	Fiber Content (%)	Fiber Length (mm)
A1	R0%	1	1.67	0.19	0.00	0%	/	/	/
A2	R20%	1	1.67	0.15	0.26	20%	/	/	/
A3	R40%	1	1.67	0.11	0.53	40%	/	/	/
A4	R60%	1	1.67	0.08	0.79	60%	/	/	/
A5	R80%	1	1.67	0.04	1.06	80%	/	/	/
B1	F9(0.2%)	1	1.67	0.08	0.79	60%	0.0086	0.20%	9
B2	F9(0.4%)	1	1.67	0.08	0.79	60%	0.0172	0.40%	9
B3	F9(0.6%)	1	1.67	0.08	0.79	60%	0.0259	0.60%	9
B4	F9(0.8%)	1	1.67	0.08	0.79	60%	0.0346	0.80%	9
B5	F9(1%)	1	1.67	0.08	0.79	60%	0.0434	1.00%	9
C1	F3(0.2%)	1	1.67	0.08	0.79	60%	0.0086	0.20%	3
C2	F6(0.2%)	1	1.67	0.08	0.79	60%	0.0086	0.20%	6
B1	F9(0.2%)	1	1.67	0.08	0.79	60%	0.0086	0.20%	9
C3	F12(0.2%)	1	1.67	0.08	0.79	60%	0.0086	0.20%	12
C4	F15(0.2%)	1	1.67	0.08	0.79	60%	0.0086	0.20%	15

**Table 5:** Requirements for a grouting material.

Test	Standard for the Experiment	Recommended Requirement
Slurry density (g/cm^3^)	JGJ/T 70-2009 [38]	≤1.9
Consistency (cm)	JGJ/T 70-2009 [38]	10–14
Fluidity (cm)	GB/T 2419-2005 [42]	16–24
Bleeding rate (%)	T/CECS 563-2018 [43]	≤3.5
Consolidation shrinkage rate (%)	T/CECS 563-2018 [43]	5
Setting time (h)	JGJ/T 70-2009 [38]	12–24
3D compressive strength (MPa)	GB/T 50266-2013 [44]	≥0.5
28D compressive strength (MPa)	GB/T 50266-2013 [44]	≥2.5

## Data Availability

Data is available upon request.
